# The Power Semantics in Self and Other Repertory Grid Representations: A Comparison between Obese and Normal-Weight Adult Women

**DOI:** 10.3389/fpsyg.2012.00517

**Published:** 2012-12-18

**Authors:** Elena Faccio, Eleonora Belloni, Gianluca Castelnuovo

**Affiliations:** ^1^Department of Philosophy, Sociology, Education and Applied Psychology, University of PaduaPadova, Italy; ^2^Psychology Research Laboratory, Ospedale San Giuseppe, Istituto Auxologico Italiano IRCCSVerbania, Italy; ^3^Department of Psychology, Catholic University of MilanMilan, Italy

**Keywords:** obesity, meanings, repertory grid test

## Abstract

According to systemic-constructivist theory, all psycho-pathological organizations are linked to specific meanings which are developed by the individual within problematic situations in the context of learning, particularly within the family. The aim of this empirical study is to support the theory that eating disorders are linked to the “power semantics,” concept developed by Ugazio. The hypothesis that the bipolar construct “winner/loser” and the associated meanings are predominant for obese people, has been verified by interviewing 44 women (22 obese/overweight; 22 controls) using the Repertory Grid Test developed by Kelly. The participants’ elicited constructs were classified according to their semantic content and the data compared using statistical techniques. The power semantics were more prevalent and important in the Obese Group than in the Control Group. These results are critically discussed, highlighting possible clinical developments.

## Introduction

The view that psychological and relational components within the familiar context are strongly involved in the development and maintaining of obesity is widespread among clinicians and researchers (Dare et al., 1990; Schmidt and Treasure, 2006; Treasure et al., 2008; Rodgers and Chabrol, 2009; Faccio et al., 2011; Faccio, 2012; Maya, 2012). Several studies have found that in families with an obese component relational systems are characterized by rigidity and inability to manage and express anger and negative emotions (Kaplan and Kaplan, 1957; Bruch, 1973; Ganley, 1989). Further researchers deepened the role of alexithymia and of emotional eating as a coping mechanisms to reduce negative emotions and anxiety, in particular those related to the relationship with others such as shame, jealousy, disappointment (Slochower and Kaplan, 1980; Goossens et al., 2009; Zeeck et al., 2011).

The communication style of families with a member suffering from eating disorders is generally characterized by stiffness, dysfunctional pattern (such as by entangled, undifferentiated relationships, or maladaptive communication), and is permeated by semantics related to control and interactional dominance (Minuchin et al., 1978; Selvini-Palazzoli, 1988; Jarman et al., 1997; Lattimore et al., 2000; Surgenor et al., 2002, 2003; Treasure et al., 2008).

The theme of power and control in obesity has been addressed by several authors in the literature (Bruch, 1973; Minuchin et al., 1978; Selvini-Palazzoli, 1981; Leitner and Grant, 1982; Button, 1985; Winter and Button, 2010) but the vast majority of empirical research on these issues has focused on other psychogenic eating disorders categories (anorexia and bulimia). Family and couple relationships with an anorexic or bulimic member seem to be characterized by the dominant themes of the control, power, and associated issues as success, competition, and shame for failure (Surgenor et al., 2002; Vidovic et al., 2005; Huke and Slade, 2006; Skarderud, 2007; Stein and Corte, 2007; McNamara et al., 2008; Romaioli et al., 2008). In addition, families with a member suffering from eating disorders are described as not being cohesive and as very conflictual, invalidating, at least in part, the psychosomatic model of Minuchin and Palazzoli (Johnson and Flach, 1985; Kog and Vandereycken, 1989; Lattimore et al., 2000; Cook-Darzens et al., 2005; Romaioli and Faccio, in press). Recent theories have also recognized the importance of the beliefs system within the family, paying attention to meanings, and attributions made by the person in relation to others (Reiss, 1981; Dallos and Aldridge, 1987; Dallos, 2004; Cook-Darzens et al., 2005; Treasure et al., 2008; Faccio, 2011).

Given the scarcity of empirical studies in the literature investigating the relevance of power theme within relationships in family with obese members and their role in developing and maintaining self and others dysfunctional attributions, this study provides an empirical research on the subject. Results might facilitate researchers and clinicians in setting programs of intervention with the obese’s family system targeted in the direction of change in self and other’s member representations; especially in those cases in which the relevance of power seems to be central.

## Theoretical Framework

The systemic-constructionist approach (Gergen, 1985; Harré, 1987; Mascolo, 1992; Ugazio and Castiglioni, 1998) gives great importance to the context of interaction in the identity construction process and it sees meanings as social products. Following this perspective, Ugazio’s (1998), Ugazio and Castiglioni (1998), Ugazio et al. (2007, 2009, 2012) theory states that the obesity (as well as all the other eating disorders) would be closely linked to the problem of meaning and to the position that everyone occupies within their context of interaction, particularly within the family. People construct their identities in interacting with others, occupying a position within the conversation. Within specific contexts of learning (particularly within the family), individuals develop a particular set of “personal meanings” (*family semantics* *polarities*) through which they give meanings to themselves and to their world. These semantic constructs are organized into polar opposites (e.g., good/bad; stupid/intelligent; right/wrong). Starting from these assumptions, psycho-pathological symptoms are linked to specific semantic meanings, characteristic of each patient’s family. Therefore psychopathology can be seen as a science of meaning.

According to Ugazio’s thesis, in obesity (as well as anorexia and bulimia) the dominant semantic dimension relates to power. This dimension can be identified with the construct “winning/losing” and other related constructs. Specifically, obese people would occupy the polarity “losing” while anorexics would place themselves close to the polarity “winning.” Since the feeling of “winning” or “losing” is considered a fundamental condition to define their identity and this positioning depends solely on interpersonal comparison, these families should be characterized by an eternal struggle to be able to gain control of the relationship.

Eating disorder should arise when power semantics become salient and pervasive among family interactions and when one of the components is unable to take a position within this dimension of meaning.

Previously, several authors (Sluzki and Veròn, 1971; Guidano, 1987, 1991; Bara, 1996; Vidotto et al., 2006; Romaioli and Contarello, 2011; Faccio et al., 2012b) have dealt with the relationship between psychopathology and meaning. The great innovation introduced by Ugazio is precisely the constructionist connotation given to the concept of meaning: the semantics dimensions are actively constructed within the family through continuous modifications and negotiations, therefore they are always open and renewable in interactions with others.

## Aims and Hypothesis

The aim of this research is to test if the “power semantics” (the construct “winning/losing” and its related constructs) is the prevalent meaning dimension among obese subjects, according to Ugazio’s systemic-constructivist theory about eating disorders (1998, 2007). The present study represents the continuation and deepening of two previous researches based on Ugazio’s family semantics polarities theory. The first study (Castiglioni et al., 2003) concerns the importance of power semantics in a group of 12 obese youths, compared to an equivalent control group. The second study (Castiglioni et al., in press) concerns the salience of the semantics of power in 30 youths with eating disorders (10 anorexics, 10 bulimics, 10 obese), compared to an equivalent control group. The surveys show that among the obese youths group, constructs relating to the semantics of power are more frequent, even though they are not always considered more important than in the control group.

Starting from these two works, the present research aims to test Ugazio’s theory among obese subjects, expanding the number of participants (from 10 to 22), and extending the investigation to a different age group (40–55 years old obese women). All the researches share the same theoretical and methodological framework. Therefore two hypotheses are formulated:

1. Obese women make a higher use of constructs that relate to the semantics of power, compared to normal-weight women.
2. Obese women consider power semantics more important than normal-weight women.

### Methodology

#### Participants

The participants were 44 women, divided into two groups of 22, according to their Body Mass Index (BMI). They were all between 40 and 55 years old (average age for each group: 49 years old), they all lived in the North of Italy and were characterized by a middle class socioeconomic background. Group A consists of 22 overweight or obese women (average BMI: 33.9); they were recruited at the Department of medical and surgical treatment for obesity, Research Centre of Padua University. The inclusion criteria were: BMI >26; obesity not being caused by metabolic, physical, or iatrogenic disorders; absence of any diagnosed psychopathology or mental retardation. Group B consists of 22 normal-weight women (average BMI: 20.4). They were recruited in primary and secondary schools, the neighborhood council, and the parish center.

The inclusion criteria were: BMI between 18 and 25; the confirmation that they have never followed diets or physical exercise regimes in order to lose weight; absence of any diagnosed psychopathology or mental retardation.

Except for the “body weight” variable, the criteria used to choose the subjects were designed to make both groups as similar to each other as possible. However, the two groups should be considered statistically independent, because it is impossible to control all the potential variables which might influence the research outcomes.

#### Instruments

All the participants signed an informed consent form and the approval by the ethical committee of Padua University was obtained. An identification schedule was used to collect personal data, anthropometric data, information on lifestyle, and profession of the participant. The schedule was filled out together with a researcher who could benefit from a brief interview to deepen any relevant aspects that might be useful for the creation of the research groups.

Once created groups, each participant carried out individually a *Repertory Grid Technique* (Kelly, 1955; Fransella et al., 2004), which is an interviewing technique for identifying the ways that a person construes (interprets/gives meaning to) his or her experience. It provides information from which inferences about personality can be made, but it is not a personality test in the conventional sense. It is underpinned by the Personal Construct Theory developed by Kelly (1955). This constructivist technique, widely used in different research contexts (Yorke, 1978; Marsden and Littler, 2000; Winter, 2003; Faccio et al., 2012a) is suitable to explore the personal meanings that the subject attributes to describe him/herself and people significant for him/her. Since the focus of this research was not the process of attribution of meaning itself, but the product of this process (the eliciting meanings), the Repertory Grid technique was considered the most appropriate instrument. Interviews were audio-recorded and transcribed with the consent of the respondents, also the compilation of the grid was made with the participant during the interview.

We followed four stages for the construction of the repertory grids:

1. The elements’ identification, using the *elicitation through discussion method* (Easterby-Smith, 1981). These elements are the most important people for the subject, including “Someone I admire” and “Someone I detest.” Other elements were already assigned by the researcher (“Self,” “Someone I admire,” “Someone I detest,” “Me as I would like to be,” “Me as others see me,” “Me as others would like me to be”).
2. The elicitation of bipolar constructs using the *dyadic method* (Epting et al., 1971; Keen and Bell, 1980) and the *cardstock technique*: each element was written on a card and presented in pairs to the subject; the subject compared elements two by two, describing what they had in common and in what they were different, in order to obtain a series of bipolar semantics constructs (e.g., good/bad, smart/stupid, good/bad…). The *laddering technique* (Hinkle, 1965; Bannister and Fransella, 1980) was applied on each construct in order to increase their number.
3. The degree to which each construct could be applied to each “element” was assessed using a scale from 1 to 7. This procedure is useful to see the positioning of each element according to the polarity of the construct; although it was not used in our data analysis, however, it was carried out for further research developments.
4. Use of the *resistance to change technique* (Fransella et al., 2004) in order to list the constructs according to a specific hierarchical order, from the most to the least important. This procedure gives qualitative information from another perspective because the subject can choose among all the elicited semantics constructs, those considered the most important.

### Data analysis

All constructs were classified by two independent judges into 27 mutually exclusive categories based on the semantic content (Green, 2004). In order to simplify the analysis 18 of these categories were subsequently grouped into two super-ordinated categories: nine classes were grouped together in the category “social relations” and nine in the category “temperament.” The new grouping criteria allowed researchers to identify 11 mutually exclusive categories.

The placement of constructs in the various categories were established taking into account the opinion expressed by two independent judges whose level of inter-rater agreement, measured by Cohen’s Kappa coefficient, was 0.967.

Due to the interest of the research to the semantics of power, according to the theory of Ugazio (1998), three categories referred to this dimension of meaning were predefined by the researchers. These three categories were constructed on the basis of semantic categories related to the power used by previous research (Castiglioni et al., 2003, in press) and those defined by Ugazio et al. (2009) for the semantic analysis of therapeutic interviews.

The three power categories predefined by the researchers were:

“Power” category includes all constructs verbalized making explicit use of the words “power,” ”winning/losing,” “will,” or their synonyms or derivates (e.g., “always wants to get his own way/lets things go”; “powerful/passive…”).
“Related to power.” This category includes all constructs expressing meanings related to “winning/losing,” “power,” and “will” but without explicit mention of these words (e.g., “headstrong/gives in easily”; “strives to stand out/keeps a low profile”; “always wants to be right/adapts…”).
“Determination” category includes constructs expressing “tenacity” and “will” which do not refer to relationship but to content (Watzlawick et al., 1967; e.g., “keeps going until it reaches what he wants/gives up …”).

Data analysis was carried out comparing Group A (obese women) with Group B (normal-weight women). In order to test the first hypothesis, which predicted that obese women would make quantitatively greater use of the power semantics, the two groups were compared for the categories “power,” “connected to power,” and “determination” (considering each category individually and taken all together). Although the constructs are nominal variables, the analysis was done using the frequency of constructs elicitation, also data were distributed on an ordinal scale. The same respondent could in fact elicit a particular construct more than one time and the researchers considered the entire elicitation range. Statistical analysis (with statistical software PASW statistics 18) was carried out using Mann–Whitney Test, a non-parametric test appropriate for ordinal variables and small samples.

In order to test the second hypothesis, which predicted that obese women would indicate the semantics of power to be qualitatively more important to them, we took into account only the three constructs considered by each participant to be the most important, as elicited via the resistance to change technique in categorical terms (target categories as present or absent). Thus 132 key constructs were identified (44 × 3) and the frequencies of the categories corresponding to these constructs were calculated using the Pearson’s Chi-Squared test to statistically compare the groups.

## Results

The following table (Table 1) shows the data concerning statistics for number of constructs produced by the two groups (clinic group and control group). The productivity constructs level between groups may be considered similar.

**Table 1:** **Productivity of the two groups: group size, mean, standard deviation, range, and total number of elicited constructs**.

Group	N	M	SD	Range	Tot
Obese women	22	11.2	2.5	8–18	247
Normal-weigh women	22	10.2	2	7–15	225

The first hypothesis predicted that obese women obese would make a bigger use of constructs related to the semantics of power, compared to normal-weight women. The Table 2 shows the absolute frequencies and percentages of constructs related to power for each group (the three power categories are considered individually and taken together). The comparison between the two groups using Mann–Whitney Test shows a statistically significant difference in the use of semantics categories like “power” (*Z* = −2.052; *p* < 0.05) and “related to power” (*Z* = −3.122; *p* < 0.05), which resulted to be more used by obese women. In the “Determination” category no statistically significant difference has been found (*Z* = −1.601; *p* = n.s.). Nevertheless, if the three semantic categories are taken together, a statistically significant difference is held (*Z* = −3.558; *p* < 0.05).

**Table 2:** **Absolute frequencies and percentages of constructs related to power for each group (Group A: obese women and Group B: normal-weight women)**.

Semantics categories	Obese women		Normal-weight women	
	Abs. freq.	Freq.%	Abs. freq.	Freq.%
Power	9	3.6	1	0.4
Related to power	29	11.7	5	2.2
Determination	11	4.4	5	2.2
Tot	49	19.7	11	4.8

In order to test the second hypothesis, which predicted that obese women would indicate the semantics of power to be qualitatively more important to them, we take into account only the three constructs considered by each participant to be the most important, as elicited via the resistance to change technique; therefore 132 constructs were examined (44 × 3). Chi-square Test was used to statistically compare the groups. Absolute frequencies and percentages of the relevant constructs are calculated, as shown in the Table 3.

**Table 3:** **Absolute frequencies and percentages of the constructs considered the most important by the participants, related to the power semantics, for each group (Group A: obese women and Group B: normal-weight women)**.

Semantics categories	Obese women		Normal-weight women	
	Abs. freq.	freq.%	Abs. freq.	Freq.%
Power	2	3.0	1	1.5
Related to power	12	18.2	0	0.0
Determination	0	0.0	0	0.0
Tot	14	21.2	1	1.5

The analysis shows a statistically significant difference only for “related to power” category (χ^2^ = 8.324; df = 1; *p* < 0.05), which was considered more important among obese women. In the “power” category no statistically significant difference was found “(χ^2^ = 1.000; df = 1; *p* = n.s.); however, if the categories “power” and “related to power” are taken together, a statistically significant difference is held (χ^2^ = 6.844; df = 1; *p* < 0.05). There are no constructs related to the category “determination.”

## Discussion

The two main research hypotheses seem to be confirmed by these findings: the semantics of power is the most used (quantitative criterion) and the power semantics also seem to be considered the most important by obese people (qualitative criterion). This difference is not confirmed for the “determination” category, then it does not refer to the tenacity and the personal will dealt in the relationship with themselves, but rather to situations in which women compare positions between winners and losers in relationships. The category “determination” had in fact been included in an attempt to limit categorization of constructs errors by the researcher, in order to restrict the semantic to the relational field. Therefore, the semantics of power is not relevant as “intra-psychic dimension” or as individual characteristic, since it assumes importance in a relational perspective.

It also has to be considered that in the present research the categories “power,” “related to power,” and “determination” were interpreted in a very restrictive way, having eliminated all constructs that could be considered just similar to those investigated. In other recent studies (Ugazio et al., 2009) authors have considered and included in the semantics of power even constructs such as “personal and social success” and “arrogance,” since they emerged from clinical interviews and self-narratives by patients with eating disorders. A fortiori then, the significance of the differences emerged between the two groups, since we adopted very selective criteria for categories related to power, provides further support of the importance of this meaning dimension in obesity. In the case of considered subjects (women with children and husband all living with the family for the both groups) it would not make sense to consider variables such as separation or divorce, while it would be more appropriate (in a further research) to consider the specificity of the couple’s relationship in terms of dominance perceived between partners.

Findings are in line with the theory of Ugazio (1998) which sustains that in people with eating disorders (including obesity) the relationship contextualizes the self. It is then perceived as central in defining themselves; communication within these families seems to be characterized by an eternal struggle for supremacy, regardless of the “content” of the conflict.

On the other hand, the “determination” category might be little used by the both groups due to the age of participants; in this sense it could be interesting a comparison with younger women (e.g., with normal-weight and obese girls). Even a comparison with and between males, youth and adult, may be useful with respect to the feeling of volition and tenacity.

Results are also in line with previous studies (Castiglioni et al., 2003, in press; Castiglioni and Veronese, 2008). These findings should not be overemphasized because of the modest sample of participants, anyway, they make possible some suggestion about self and others representations in obese women. As it is well known, often the refusal of food (typical for anorexic individuals) is a means to communicate superiority, to demonstrate tenacity, and will power in order to “win” the challenge to self-determination with the other members of the family; on the contrary to surrender the impulse of hunger is equivalent to declare to be losers in comparison to everyone who is able to control himself. In this sense, it would be interesting to investigate the self representation in terms of positioning. Castiglioni et al. (2003) found out that obese people tend to position themselves into the negative pole of the construct winner-loser and tend to describe the self as passive and resigned.

This description corresponds to the stereotype of the obese as a harmless person, humorous but also passive, weak, and submissive (Enzi, 1997; Molinari and Riva, 2004). A positioning of this kind could be also attributed to the “internalization” of the stereotype (Kalish, 1979 in Cacciaguerra, 1992) by side of the obese. On the basis of these observations it would be interesting to study further the relationship between common-sense stereotypes attributed to the obese and what self-representations obese people build.

Lastly it would be useful to redefine power semantics categories using other semantic criteria as meanings expressed by patients suffering from eating disorders during therapeutic conversation or self-narratives (Ugazio et al., 2009).

## Conclusion

It would be interesting to develop this study toward multiple directions: to deepen the investigation into Repertory Grids taking into account the positioning of each subject; to increase the number of participants or extend the investigation to a group of obese men (to have comparison between sexes); to widen the investigation, comparing the results with other methods (e.g., analysis of therapeutic discussions).

This work suggests also some reflections on a clinical level. Therapy could be aimed at co-constructing new and different meanings in order to develop a more wide and multidimensional construct system.

Given that the construction of self is intrinsically related to the construction of others, it will be important to develop patient’s construing of other people trying to look at the world from another perspective. Such exploration can take many different forms, one way is, for example, the fixed role therapy (Kelly, 1955). In conclusion, the use of Repertory Grids in a clinical context provides the clinician with a map of patient’s salient dimensions of meaning and positions adopted by him/her and by other family members. The repertory grids may also be useful in monitoring the therapeutic intervention, showing changes about how the patient places him/herself in relation to the critical dimension and showing the emergence of new meanings (Faccio et al., 2012a). A simple comparison between initial and final grid data provided at the end of therapy by the same person may represent also a valid help for evaluating the efficacy of psychological therapy in modifying self-representations. In this way it will be possible to place the power theme into a more appropriate context (e.g., work, sport, etc.) rather than being constantly at center stage of family system. This consideration may also be useful and not orienting also clinical session with obese members family, favoring a deeper awareness of importance given to dominance or passivity in relationships between members, and helping them in discovering different way for representing self and others.

Clinical psychology has to address obesity developing specific protocols and models (Manzoni et al., 2008; Castelnuovo, 2010a,b; Pietrabissa et al., 2012) above all in new settings (Molinari et al., 2012) and technology-based scenarios (Castelnuovo et al., 2003a,b, 2010, 2011a,b,c; Castelnuovo and Simpson, 2011; Manzoni et al., 2011), improving not only evidence-based prescriptions (Castelnuovo et al., 2004, 2005, 2008; Castelnuovo, 2010b), but also best practices such as the applications related to the promising Ugazio’s theory in analyzing psychological processes in female obesity.

## Conflict of Interest Statement

The authors declare that the research was conducted in the absence of any commercial or financial relationships that could be construed as a potential conflict of interest.
